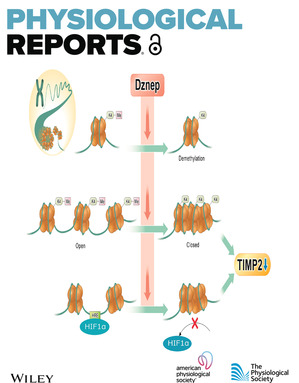# Cover Image

**DOI:** 10.14814/phy2.15846

**Published:** 2023-12-15

**Authors:** Tomotaka Yamazaki, Imari Mimura, Yu Kurata, Tetsuhiro Tanaka, Masaomi Nangaku

## Abstract

The cover image is based on the Original Article *Dznep, a histone modification inhibitor, inhibits HIF1α binding to TIMP2 gene and suppresses TIMP2 expression under hypoxia* by Tomotaka Yamazaki et al., https://doi.org/10.14814/phy2.15810